# Gaylord and Aschoff’s Pathological Histology

**Published:** 1902-04

**Authors:** 


### BOOK REVIEWS.

---

GAYLORD AND ASCHOFF'S PATHOLOGICAL HISTOLOGY. The Principles of Pathological Histology. By HARVEY R. GAYLORD, M. D., Professor of Surgical Pathology in the University of Buffalo, N. Y.; and LUDWIG ASCHOFF, M.D., Professor and First Assistant in the Pathological Institute of the University of Göttingen, Germany. With an introductory note by William H. Welch, M. D., Professor of Pathology in Johns Hopkins University, Baltimore. In one quarto volume of 354 pages, with 81 engravings in the text and 40 full-page plates. Philadelphia and New York: Lea Brothers & Co. 1901. [Price, cloth, $7.50 net.]

In the preface to this work the authors state that "it is intended to supplement the regular course in pathological histology and to lead the student and graduate to the application of such knowledge in practice." The difficulty of conveying a correct conception of pathological histology outside of a laboratory and apart from actual sections of various diseased conditions, is so great that much difference of opinion exists as to the best mode of presenting the subject in a textbook. This work does not in any way pretend to be a textbook, but rather an atlas covering the principles of a course in practical pathology, and the authors have attempted to atone for the lack of actual demonstrations by presenting, by means of the heliotype process, a large number of photomicrographs of various pathological conditions.

This work has only recently appeared in German and its present appearance in English must be gratifying to all native readers. The German edition presented a high standard, but the English shows a decided improvement thereupon. The plates, instead of being bunched together in the rear of the book, are distributed throughout the text, making them more accessible and impressive. In our opinion, they are the very best yet produced in connection with similar medical works. The skill of Dr. Gaylord, one of the authors, in connection with microphotographic reproductions, is proverbial and he has here given us just what was natural to expect from an expert. The volume, as a whole, surpasses all others that have thus far been published—primarily in regard to the plates, which clearly demonstrate what can be done in the line of reproductions of this kind, especially when the publishers hesitate at no expense which will help to insure an intelligent appreciation of their most excellent pictures. In addition to the illustrations in black, we find thirty-five colored ones, representing various abnormal changes, which, either for their color correctness (amyloid) or their natural tint (pigment), deserve special mention. The authors show what can be accomplished in the way of tricolor photography, by giving a number of reproductions of this most recent art. These were obtained through the microscope, the color plates being made from color negatives taken directly from stained preparations. The result is satisfactory, considering the newness of the method, although the usual defects are still in evidence—the lack of sharpness in outline and the blending and blurring of colors. Opposite every plate a brief explanation is given and the reader is directed to the particular page where fuller information may be obtained.

The general arrangement of the book is in harmony with the well-known methods of Professor Orth, to whom this volume is dedicated. It is no easy task to prepare a work of this kind, to describe in full the pathology peculiar to each organ and the conditions of the various diseases, especially in this era of progressive medicine when about every month is marked by the proposition of some new theory, followed by various conjectures as to its etiology and pathology.

The work comprises 364 pages and is divided as follows:

Part I., microscopical technique; part II., pathological histology of organs; part III., the principles of optics and photomicrography.

In the first few pages, brief instructions are given regarding the use of the microscope, also for the manipulation of fresh material applicable to the investigation of fluids and tissue, which of late is considered very important in view of the fact that the old methods changed the tissue, while now it can be examined when fresh. The subject of histological technique is quite fully discussed—it would, of course, be impossible to include all the numerous suggestions; however, the omission of Stephanow's rapid collodion method is sure to be noticed. The section on the technique of embedding and staining is valuable, although neither full nor complete. Still many of the methods eliminated are wholly unnecessary, having been superceded by more modern modes.

Taken as it is, this work will enable one with a fair knowledge of laboratory technique to make a thorough examination of the latest and most improved methods.

The second part constitutes the principal portion of the book; gives a remarkably full epitomé of the pathological histology of organs, preceded by a proper reference to the normal, and followed by a concise description of the pathological changes of the various organs of the body. All this is admirably set forth. It is, of course, impossible in a review so brief as this to lay special stress upon all the good features of the work; let it be said, however, that the text on pathological lesions could hardly have been better, or done with fewer errors in description. All the chapters of the second part give proof of the author's intimate and practical acquaintance with pathology. The various tumors are clearly described and special attention is given to the etiology of carcinoma. However, this portion of the book does not cover as much ground as might have been expected, considering that one of the authors has made this subject a special study and is a recognised authority concerning some of its phases. The author, of course, confines himself to the parasitic theory, to which the attention of the whole world is at this moment directed. The bibliography relating to the subject under discussion adds greatly to the usefulness of the book.

The chapter on diseases of the blood is written by Dr. Lyon, and, considering its length, it must be claimed as one of the best, concise articles upon this subject that has yet appeared. It is written from a strongly individual point of view, possesses decided freshness of style and admirably condenses knowledge gleaned from various sources which is both interesting and valuable.

The third section is devoted to the principles and optics of photomicrography apparatus, and is very important in a pathological sense—an importance clearly shown by the attention paid by Dr. Gaylord throughout his work to photographic reproductions. He begins by fully explaining the scientific principles of optics, a matter of great moment and a subject hard to master, and so confers upon all his readers what is of real and practical benefit. Full directions for putting together a photographic apparatus are given and illustrations furnished of appliances, many of which have been contrived by Dr. Gaylord himself, whose mechanical genius is well known.

A valuable portion of this section is devoted to the steps necessary to be taken in making a picture. While the directions are not new, they nevertheless relate to what is most practical for the amateur. The authors claim that the wet-plate process is the best for making a lantern slide; this involves more detail than any other known method—a fact which the authors should have recognised. It would have been better to have given full and specific directions upon this important point. We think reference should also have been made in this connection to the accepted merits of Paget's dry plates. This portion of the work will be specially welcomed as coming at a time when it is greatly needed.

The usual errors incident to a first edition are strongly in evidence throughout the book, a drawback, which of course, will be remedied in later editions.

In conclusion, it only remains to extend hearty congratulations to the authors upon the appearance of this admirable work, which is surely destined to occupy a leading position among publications of its class.

